# Supplementary figures and images for: Bacterial community shifts of commercial apples, oranges, and peaches at different harvest points across multiple growing seasons
Source: PLoS One. 2024 Apr 16;19(4):e0297453. doi: 10.1371/journal.pone.0297453 (PMC11020611; doi:10.1371/journal.pone.0297453)

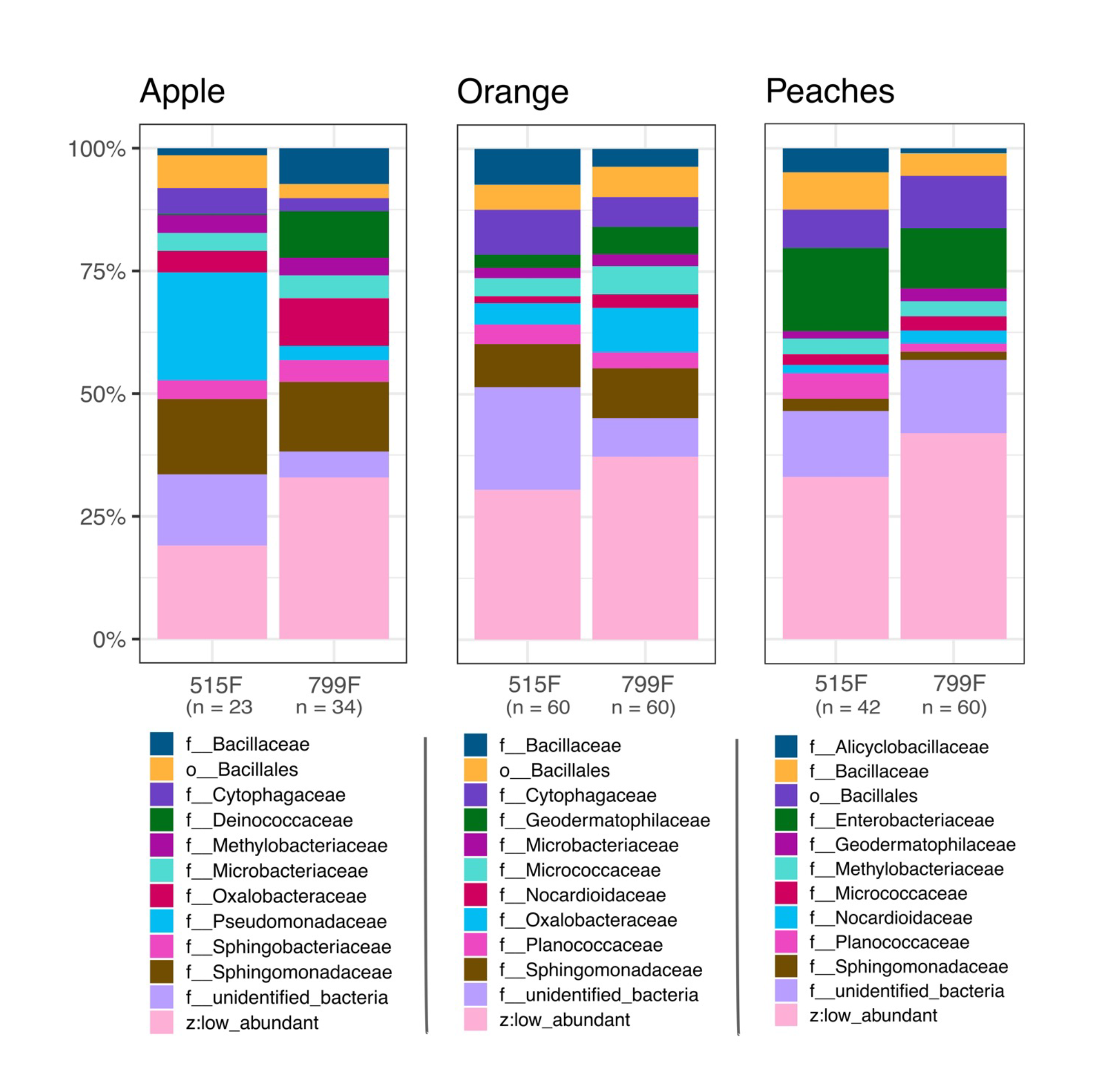

Supplement: S1 Fig — Sequences were rarefied by random permutation to 2000 sequences per sample. Note that colors should only be compared within a fruit type. Unidentified bacteria were classified only to the Kingdom level by RDP. Low abundant bacteria are bacteria taxa below the top 10 most abundant taxa at the family level, for color reasons. (TIF) [file pone.0297453.s001.tif]

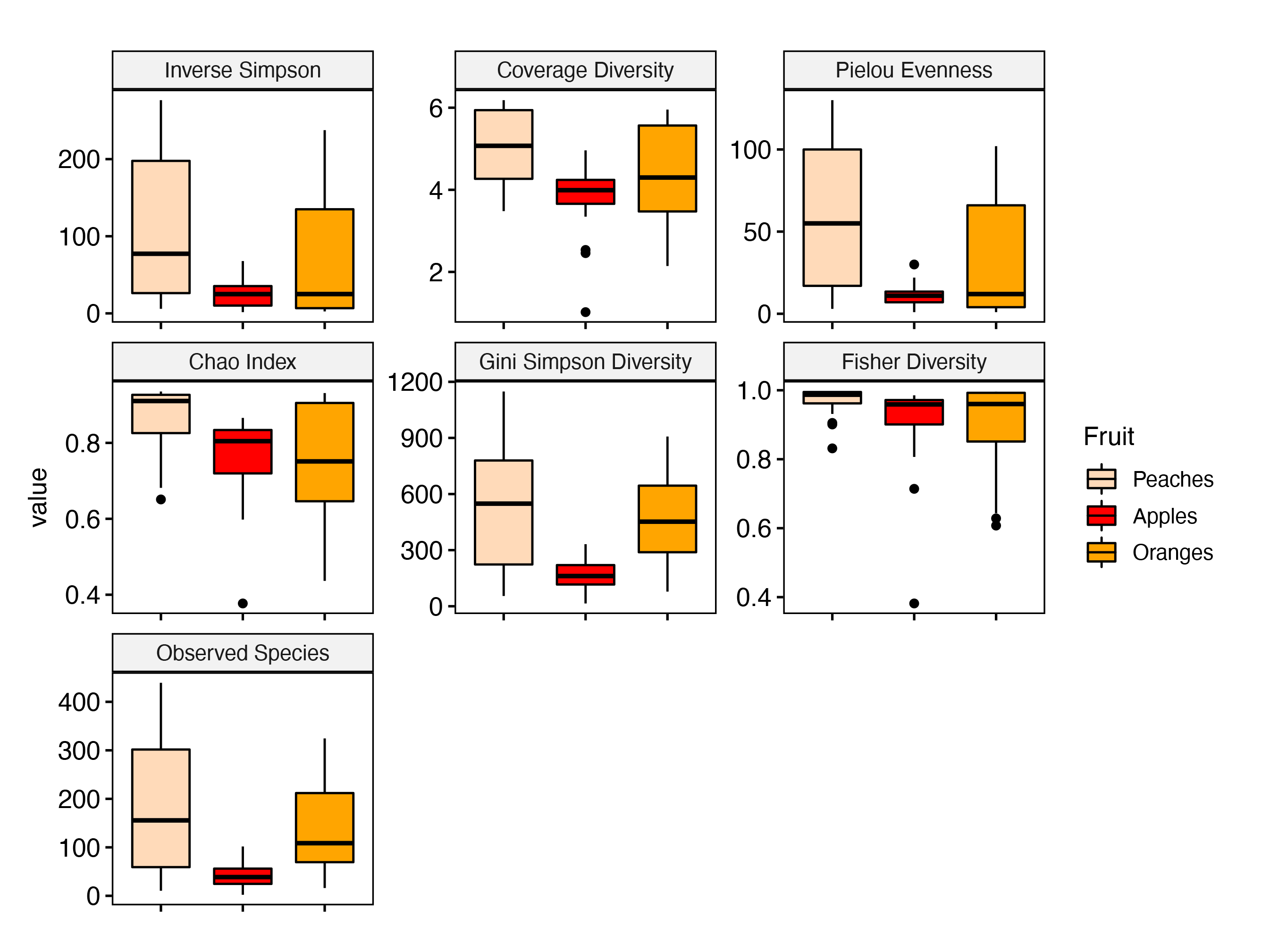

Supplement: S2 Fig — Comparing the total bacterial communities of the carposphere of the tree types of the tree fruit used in this study with different alpha diversity metrics. (TIF) [file pone.0297453.s002.tif]

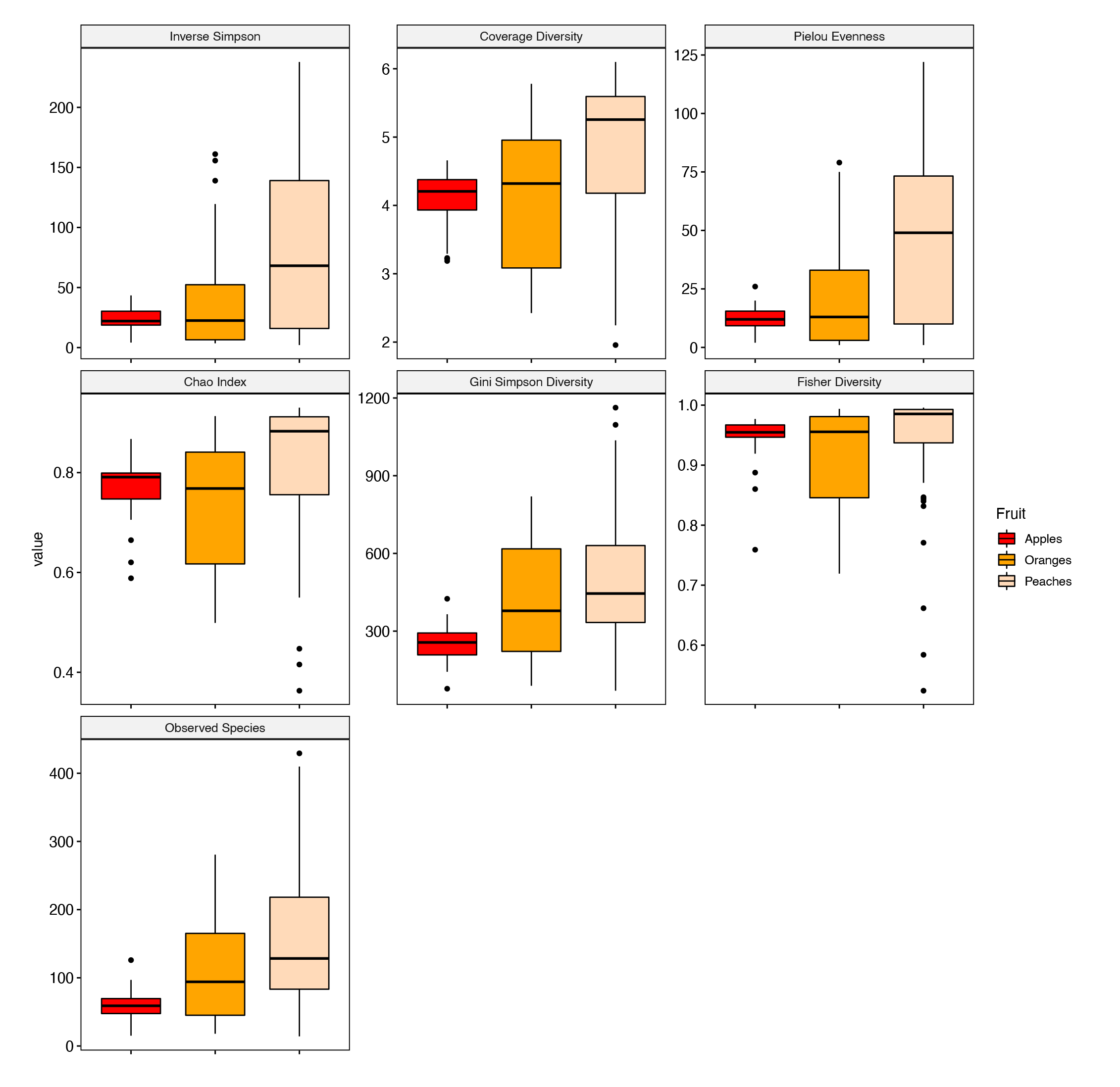

Supplement: S3 Fig — Comparing the viable bacterial communities of the carposphere of the tree types of the tree fruit used in this study with different alpha diversity metrics. (TIF) [file pone.0297453.s003.tif]

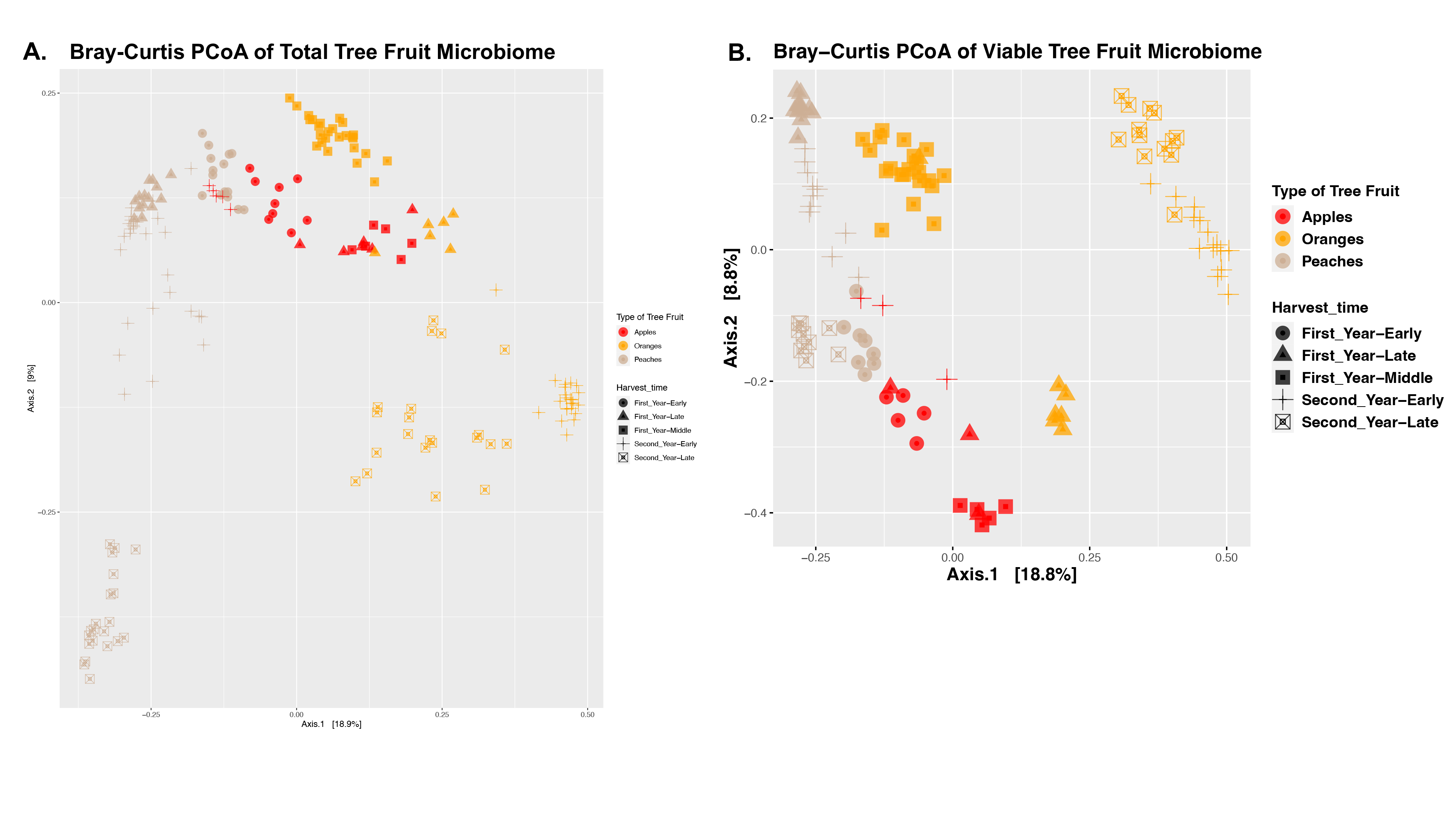

Supplement: S4 Fig — (A) Beta diversity of all the total carposphere samples for the three types of tree fruit used in this study. (B) Beta diversity of all the viable carposphere samples for the three types of tree fruit used in this study. (TIF) [file pone.0297453.s004.tif]

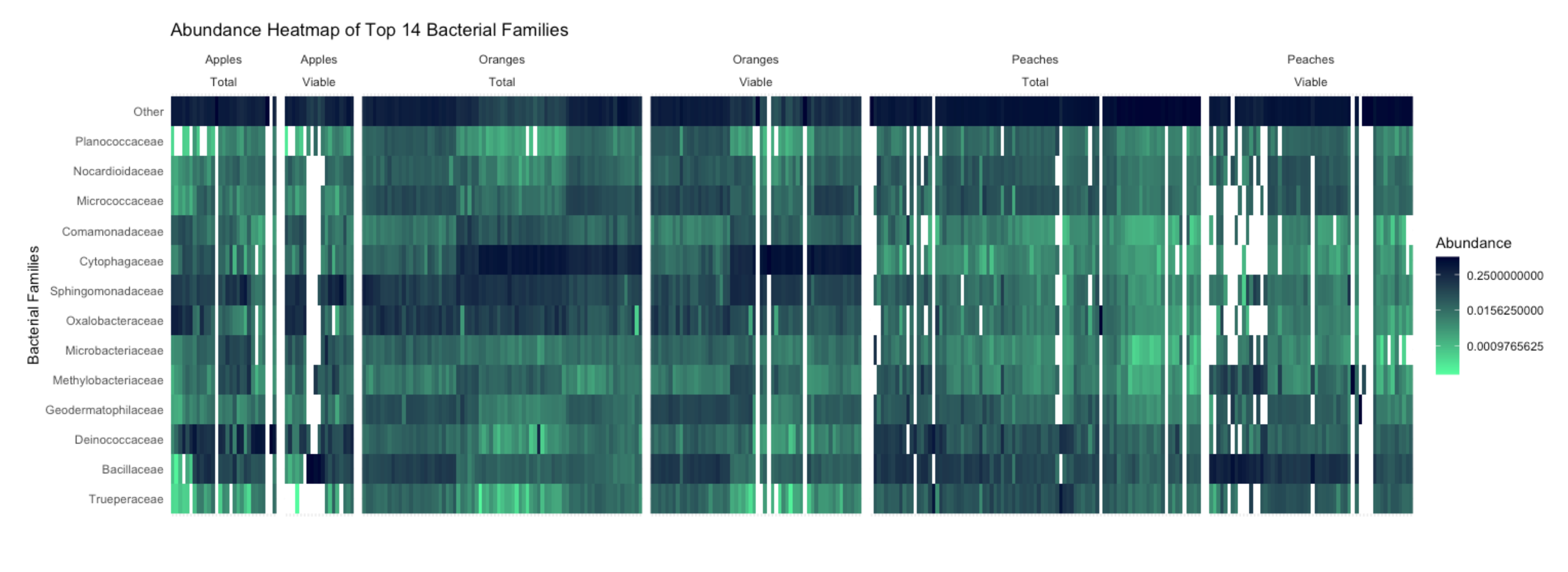

Supplement: S5 Fig — Heat map of the abundance of the top 14 bacterial communities for the three tree fruit types, further split by PMA treatment. As color darkens, the more abundant that bacterial family is in the sample. Detection was at 0.1% and prevalence of 75% were used, same as the core microbiome analysis. All samples are represented as total (non-PMA treated) and viable (PMA treated) for the respective fruit. The color scale represents the abundance of each of the top 14 bacteria present for each sample collected, going from light green to dark green. (TIF) [file pone.0297453.s005.tif]
