# Supplementary material for: Bacterial community shifts of commercial apples, oranges, and peaches at different harvest points across multiple growing seasons
Source: PLoS One. 2024 Apr 16;19(4):e0297453. doi: 10.1371/journal.pone.0297453 (PMC11020611; doi:10.1371/journal.pone.0297453)
Supplement: Attachment — Submitted filename: Response to reviewers.docx [file pone.0297453.s006.docx]

**Response to reviewers:**

***We thank the reviewers for their kind comments and excellent insights into improving our manuscript prior to publication. We have addressed all the comments in the revised manuscript and the changes for each specific comment are highlighted below.***

Reviewer #1: The study, titled "Bacterial Community Shifts of Commercial Apples, Oranges, and Peaches at Different Harvest Points Across Multiple Growing Seasons," sets a clear focus on understanding the shifts in bacterial communities associated with commercially significant fruits. The primary goal of this research was to establish a foundational profile of bacterial communities present on apples, peaches, and Navel oranges at the moment of harvest. This is a critical juncture in the life cycle of these fruits, as it marks the point at which they transition from the tree to the postharvest handling and processing stages. The study's findings hold substantial importance as they offer a comprehensive view of the temporal dynamics of bacterial communities on major commercial tree fruits. This temporal aspect adds depth to our understanding of the microbial ecology associated with these fruits. By examining the bacterial communities at harvest, the study provides valuable insights into how these communities might influence the postharvest packing and processing stages. Understanding these dynamics is crucial for ensuring the quality and safety of the fruits as they move through the supply chain.
The data is presented with meticulous attention to detail, demonstrating a thorough and comprehensive approach to the research. This not only adds credibility to the findings but also enhances the overall quality of the paper. The clarity and coherence of the writing contribute significantly to the paper's overall impact. The study makes a noteworthy contribution to the existing literature in this field. By providing a detailed analysis of bacterial communities in commercially relevant fruits, it fills a crucial gap in our understanding of fruit microbiology. Based on the quality of research, the thoroughness of data presentation, and the significance of the findings, I believe this study holds substantial merit and should certainly be considered for publication. It not only advances our knowledge in this specific area of research but also has broader implications for the fruit industry and food safety practices.

***We thank Reviewer 1 for their very kind words and appreciation of the amount of hard work that went into the study, and that they see substantial merit to the research and the publication. We agree that it will have a strong impact on the postharvest packing and processing for the tree fruit industry.***

Reviewer #2: The manuscript describes a study that examines the bacterial communities of apples, oranges, and peaches at different harvest points across multiple growing seasons. The work appears to be well-designed and the results are presented in a clear and organized manner. The findings of the study have the potential to contribute to our understanding of the bacterial communities associated with these fruits and their changes over time.

***The authors also thank Reviewer 2 for their positive feedback on the study and publication, we really appreciate the guidance to making the manuscript stronger and have highlighted the changes made to each of the comments below.***
1. The abstract is well-written and concise, but it could be more informative and specific about the main findings and implications of the study. For example, you could mention the names of the dominant bacterial taxa or families in each fruit type.

***We thank the reviewer for suggesting the addition of more specific findings to improve the abstract. We have added the core microbiome bacterial families found on each type of tree fruit to the abstract and what this means to the results of the study.***

2. The introduction could be more concise and focused on the main research question and objectives.

***We have made the introduction more concise, eliminating several points that do not directly focus ont eh main research question of the study. Therefore, the introduction is overall now more focused on setting up for our specific objectives of the study.***

3. The introduction should clearly state the problem or question being addressed in the study. The authors have mentioned that they wanted to explore the bacterial communities of apples, oranges, and peaches at different harvest points. However, a more specific statement of the problem, such as "How do the bacterial communities of these fruits change over time?", would be more engaging and would provide a clearer focus for the reader.

***We have added the specific problem that the study was looking to address to the introduction as recommended by the reviewer.***

4. I am confused by the sample size mentioned in the manuscript. According to the author's description, 50 fruits were collected from each tree, and 10 or 5 fruits were combined into one composite sample. How many trees were collected in total? How many samples in each composite sample? The author provided the number of sequenced samples in Table 1, but it does not match the number shown in Supplemental Figure 4. Therefore, the author needs to clarify the process of determining the sample size in the Methods section.

***We understand the confusion of the reviewer, and have changed the wording of the sample collection section of the materials and methods section to make it clearer to the reader. We have addressed all the concerns including how many trees were collected and how many samples in a composite sample. The difference in the numbers of Table 1 and the Supplemental Figure 4 is due to the elimination of some samples during rarefication to 2000 reads, which has been clarified in the methods section too.***

5. L167: What do you mean by commercial orchards? L167-168: Why did you choose these orchards? Any rules in choosing the geographical location?

***We have added a statement about the definition of commercial orchard to the methods section, and also a statement about why these orchards were chosen for the study.***

6. The discussion should acknowledge any limitations of the study, and provide some suggestions for future research in the field.

***We have added a paragraph at the end of the discussion that addresses the limitations of the study and the future work based on our study.***
